# The expression and clinical significance of HERC4 in breast cancer

**DOI:** 10.1186/1475-2867-13-113

**Published:** 2013-11-14

**Authors:** Hui Zhou, Rong Shi, Min Wei, Wen-Ling Zheng, Jue-Yu Zhou, Wen-Li Ma

**Affiliations:** 1 Institute of Genetic Engineering, Southern Medical University, Guangzhou 510515, China

**Keywords:** HERC4, Breast cancer, Tissue microarray, Histopathological grade, Clinical stage

## Abstract

**Background:**

Increasing evidence suggest that ubiquitin-proteasome system (UPS) plays a key role in tumorigenesis. HERC4 is a recently identified ubiqutin ligase. However, the expression status and biological functions of HERC4 in cancers are not clearly.

**Methods:**

We evaluated the HERC4 expression in breast cancer cell lines and breast tumor tissues by quantitative real-time PCR and western blot analysis. To investigate the clinicopathological significance of HERC4, immunohistochemistry analysis for HERC4 was performed on a tissue microarray including 13 benign fibroadenoma, 15 intraductal carcinoma, 120 histologically confirmed invasive ductal carcinoma. Receiver operating characteristic (ROC) analysis was applied to determine the optimal cut-off score for positive expression of HERC4, when HERC4 positive expression percentage was above 60%, tumor was defined as “positive”.

**Results:**

HERC4 was up-regulated in breast cancer cell lines and breast tumor tissues compared to non-tumorigenic cell line and adjacent normal breast tissues. According to ROC analysis, HERC4 positive expression was detected in 1/16 (6.3%) of normal breast tissue, in 3/13 (23.1%) of fibroadenoma, in 6/15 (40%) of intraductal carcinoma and 66/120 (55%) of invasive ductal carcinoma. Positive expression of HERC4 was positively correlated with pT status, pN status, clinical stage and histological grade of patients with invasive ductal carcinoma (p < 0.05).

**Conclusions:**

Our findings suggest that HERC4 was a significant diagnostic marker for invasive ductal carcinoma of the breast.

## Background

Breast cancer has been the leading cause in cancers that threat global women life and health [1]. Breast cancer has the characteristics of high incidence and mortality rates. About 1.3 millions women will be diagnosed with breast cancer and 0.45 millions deaths are estimated in 2013 according to GLOBOCAN. Different types of treatment are performed for patients with breast cancer due to heterogeneity [2,3]. Surgical resection is effective therapy for early or local cancer, however, this method may be not suitable when cancer metastasis occurred [4]. Radiation therapy, chemotherapy, hormone therapy improve the survival time to some extent, but accompanying pain, resistance and side effects can not be ignored [5,6]. Breast cancer targeted therapy is a more accurate and specific treatment that uses certain drugs or other substances to identify and target cancer cells without harming normal breast cells. In clinically, estrogen receptor (ER), progesterone receptor (PR), and human epidermal growth factor type 2 receptor (HER2) are established biomarkers for breast cancer diagnosis, cancer subtypes analysis and specific treatment guidance. Furthermore, targeted therapeutics for breast cancer are mainly concentrated on HER2 which is tested as overexpression in 20%- 25% of invasive breast cancers [7]. Trastuzumab and lapatinib are used as the anti-HER2 first-line drugs for HER2-positive patients [8]. On the one hand, The overexpression of HER2 is closely related to poor prognosis and disease-free interval [9]. On the other hand, targeted therapeutics against HER2 are more appropriate treatments for metastatic breast cancer patients with HER2-postive and efficacy will be discounted for triple-negative breast cancer [8]. Combined with drug resistance, these results promote more novel targets to be identified for diagnosis, prognosis and targeted therapies.

The mechanisms of molecular tumorigenesis and development of breast cancer remain unknown. In general, breast cancer is closely associated with disorder of gene expression, gene mutation and destruction of protein homeostasis [10]. Ubiquitin-proteasome system (UPS) plays a crucial role in the dynamic balance of intracellular proteins which is different from non-selective protein degradation by lysosome [11]. Ubiquitin is a ubiquitous and conserved protein composed of 76 amino acid. Ubiquitin-proteasome pathway begins with the attachment of ubiquitin to selected protein [12]. The sequential biochemical reactions involve three ubiquitin-related enzymes: ubiquitin activating enzyme (E1 enzyme), ubiquitin conjugating enzyme (E2 enzyme) and ubiquitin ligase (E3 ligase). At last, proteins tagged with ubiquitin are transported into the 26S proteasome for specific degradation. Moreover, among the ubiquitin-related enzymes family, ubiquitin activating enzyme is unique and the number of ubiquitin conjugating enzyme is restricted. However, there are numerous ubiquitin ligases which determine the specificity of substrate recognition [13]. The normal operation of this mechanism ensure various life activities, such as cell cycle, apoptosis, DNA damage response, signal transduction, et. al. If abnormality happens, it would lead to the occurrence of diseases, including cancer [14]. Growing evidences suggest that ubiquitin-proteasome system holds an important status in etiology of cancer [15,16]. Consequently, ever-increasing studies focus on ubiquitin ligases which specifically recognize substrate protein.

HERC4 is a member of HERC family which is characterized by HECT domain and at least one RCC1 (regulator of chromosome condensation 1)-like domains (RLD). HERC protein has a dual function that RLD perform the function of guanine nucleotide exchange factor (GEF) for Ran like RCC1 and HECT plays a role of ubiquitin ligase [16,17]. HECT domains have about 50% similarity to carboxyl-terminal region of E6-associated protein (E6AP) which possesses ubiquitin ligase activity for p53 [18]. Karin et al., identified HERC4 firstly and defined HERC4 as the common ancestor of HERC family by phylogenetic tree analysis [19]. Immunofluorescence showed HERC4 localize to the endosome and lysosomes [20]. Murine HERC4 play a key role in spermatogenesis mediated by loss of proteins and organelles via ubiquitin-proteasome pathway. Inactivation of murine HERC4 would influence spermatozoon maturation and consequently results in decrease in male fertility [21]. However, in human cancer biology, the physiological substrates and biological functions of HERC4 are unknown. Therefore, our aim in this study was to investigate whether HERC4 play a role in the development of breast cancer and clinical significance. Here we report that HERC4 was up-regulated in tumor cells of clinical breast cancer samples. The expression of HERC4 in invasive ductal carcinoma was positively related to the clinical stage, histological grade and pT status. Moreover, HERC4 was increasingly expressed in patients with lymph node metastasis.

## Results

### Protein expression of HERC4 in breast cancer cell lines

**Figure 1:**
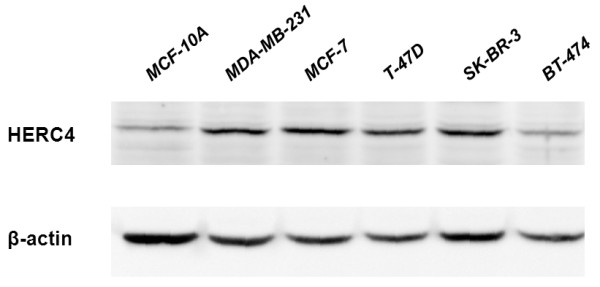
**Western blot analysis of HERC4 expression in breast cancer cell lines.** β-actin was used as internal control.

To determine whether expression difference of HERC4 exist between non-tumorigenic cell line and tumorigenic cell lines, we analysed HERC4 expression in one non-tumorigenic breast epithelial cell line (MCF-10A) and five tumorigenic breast epithelial cell line (MCF-7, MDA-MB-231, T-47D, SK-BR-3 and BT-474) by western blot (Figure 1). Interestingly, low expression of HERC4 in MCF-10A and high expression in other tumorigenic cell lines except for BT-474 were found. This suggested that breast cancer cells may have a higher expression level of HERC4.

### Protein expression of HERC4 in 32 breast cancer tissues compared to adjacent nontumor breast tissues

**Figure 2:**
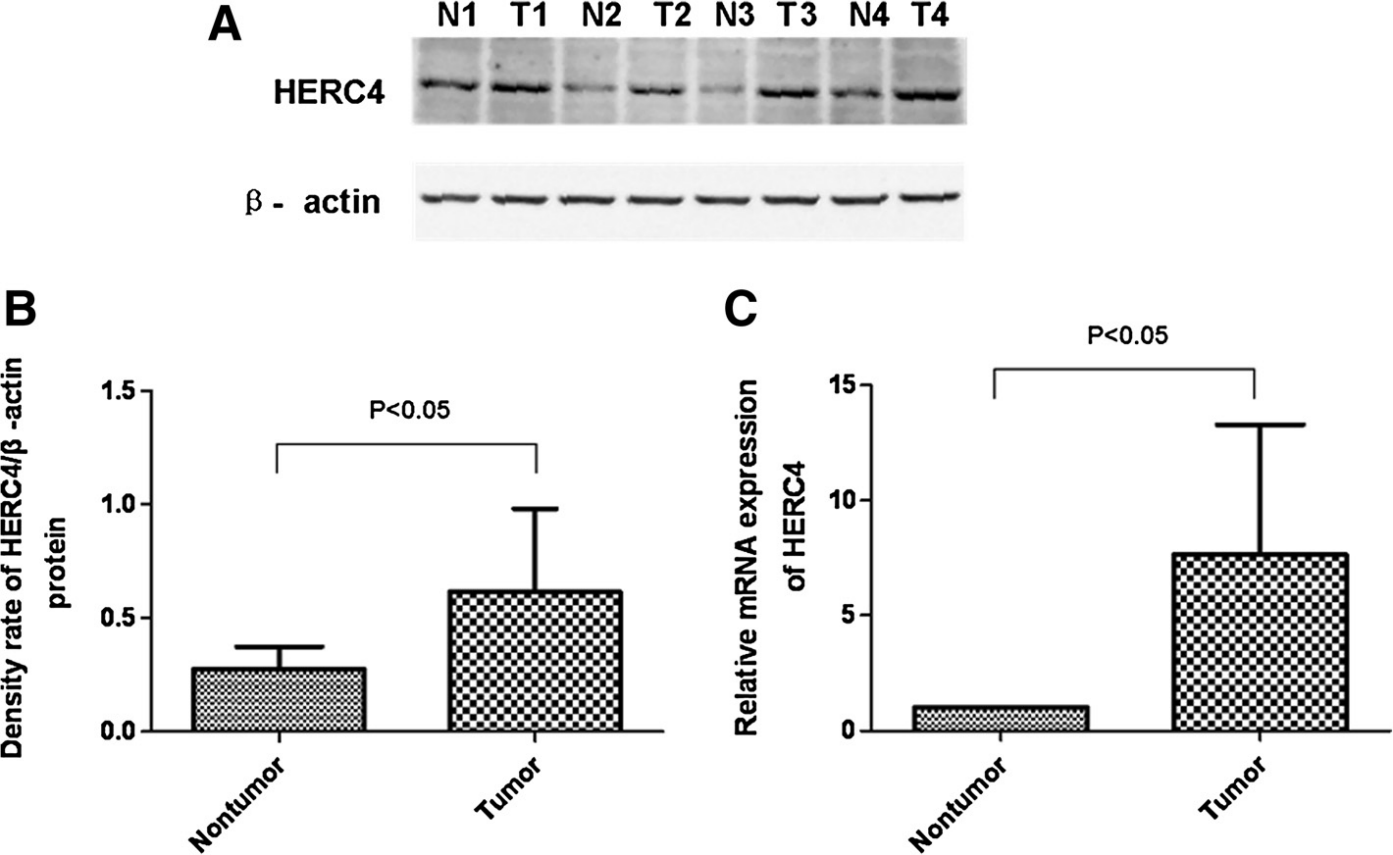
**Real-time PCR and western blot analysis of HERC4 expression in 32 paired breast cancer and adjacent normal breast tissues. (A)** Western blot indicated significant up-regulation in breast cancer tissues (T1, T2, T3, T4) in comparison with in the adjacent nontumorous breast tissues (N1, N2, N3,N4). β-actin was used as internal control. **(B)** Western blot was calculated as optical density value and expressed graphically. Significant differences of HERC4 protein expression between tumor (T) and adjacent nontumorous tissues (N) were analyzed statistically using the ratio between the optical densities of HERC4 and β-actin. HERC4 protein expression was significantly higher in breast cancer tissues (P < 0.05). **(C)** Significant differences of HERC4 mRNA level between tumor (T) and adjacent nontumorous tissues (N) were analyzed statistically by 2^-ΔΔct^ method. HERC4 mRNA expression was significantly higher in breast cancer tissues (P < 0.05). GAPDH was used as internal control.

Real-time PCR and western blot analysis were used to detect HERC4 expression in 32 paired breast cancer and adjacent normal breast tissues. Similarly, high expression of HERC4 was founded in clinical breast cancer tissue in comparison with adjacent normal breast tissues. The western blot result of four paired samples was showed in Figure 2A and the value of optical density of the tumorous (T) and nontumorous (N) tissues was graphically expressed in Figure 2B. The mRNA expression detected by quantitative real-time PCR was showed in Figure 2C. The result showed that expression level of HERC4 mRNA was about 6-folds elevated in breast cancer tissues compared with adjacent normal breast tissues.

### Cut-off score selection and immunohistochemical expression of HERC4 in tissue microarray

**Figure 3:**
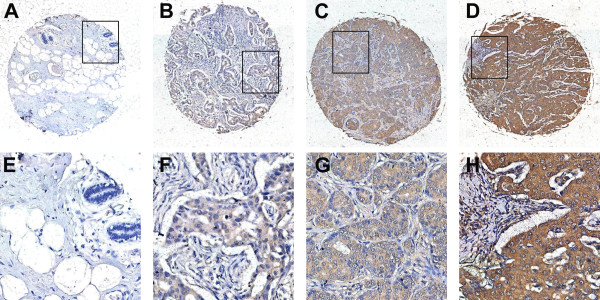
**The expression of HERC4 in adjacent normal breast tissue and breast cancer tissues by IHC. (A)** Negative expression of HERC4 was detected in normal breast tissue (100x). **(B)** Negative expression of HERC4 was detected in an invasive ductal carcinoma (case 57), in which less than 30% of tumor cells showed positive staining (100x). **(C)** Positive expression of HERC4 was showed in an invasive ductal carcinoma (case 89) with 80% staining extensity and moderate intensity (100x). **(D)** Positive expression of HERC4 was showed in an invasive ductal carcinoma (case 105) with 95% staining extensity and strong intensity (100x). **(E)**, **(F)**, **(G)** and **(H)** demonstrate the higher magnification (400x) from the area of black box in **(A)**, **(B)**, **(C)** and **(D)**, respectively.

**Figure 4:**
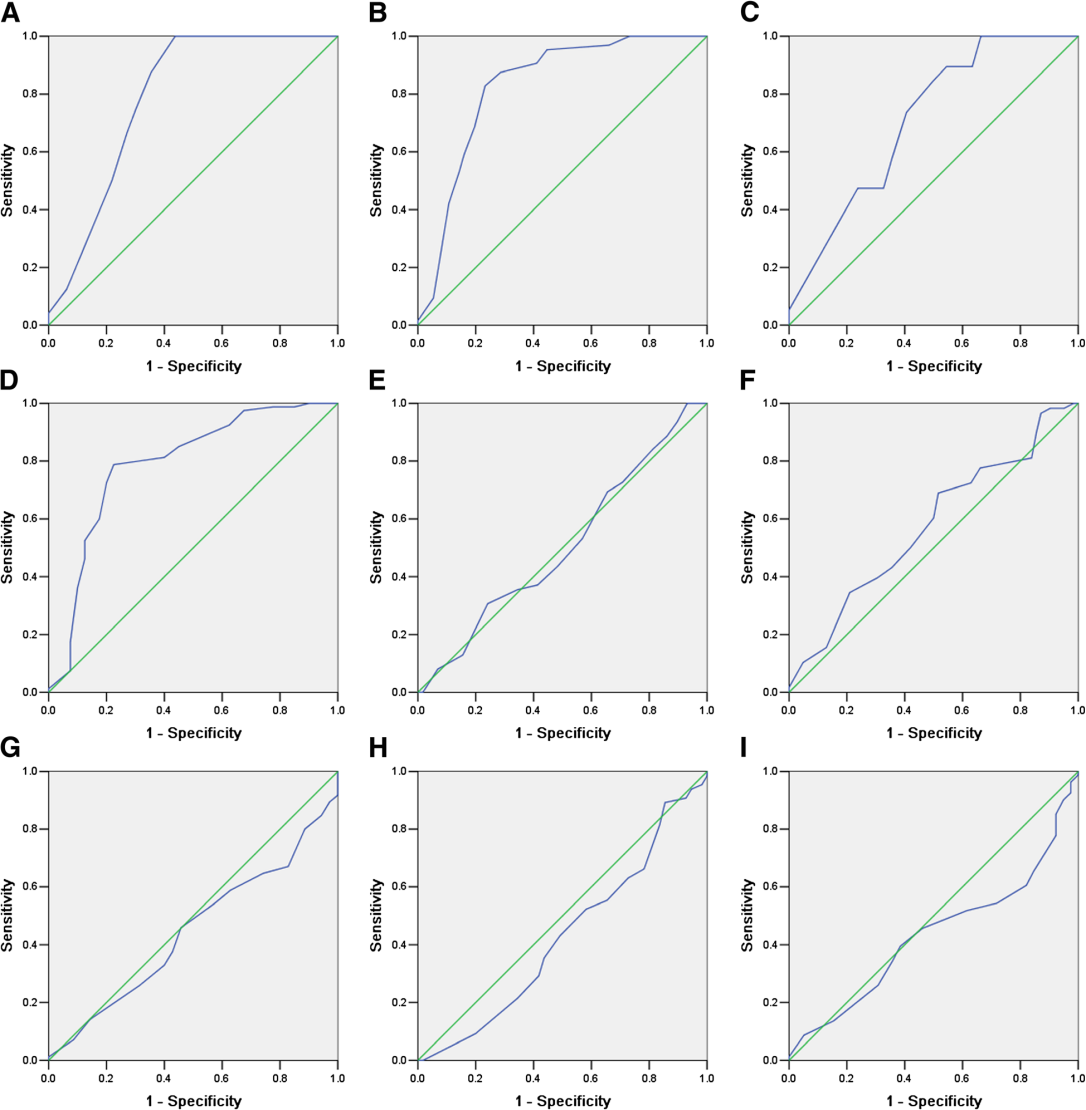
**Receiver operating characteristic (ROC) were applied to select cut-off score for positive expression of HERC4.** Various ROC curves were plotted by sensitivity and specificity for each outcome: **(A)** histological grade, **(B)** clinical stage, **(C)** pT stage, **(D)** pN stage, **(E)** ER status, **(F)** PR status, **(G)** Her2 status, **(H)** p53 status, **(I)** Ki67 status.

IHC was conducted to investigate the expression pattern of HERC4 in breast cancer and normal breast tissues. Immunoreactivity was observed primarily in cytoplasm of tumor cells and IHC staining for HERC4 in representative samples of breast tumor and normal breast tissues were shown in Figure 3. Optimal cut-off score for HERC4 was derived from ROC analysis (Figure 4). In our present study, ROC curve analysis for clinical stage showed that the point on the curve had the shortest distance to the point (i.e. 0.0,1.0). Thus, we selected the cut-off score according to clinical stage and defined tumor as HERC4 positive when HERC4 expression percentage was above 60%. On the basis of cut-off score, HERC4 positive expression was detected in 1/16 (6.3%) of normal breast tissue, in 3/13 (23.1%) of fibroadenoma, in 6/15 (40%) of intraductal carcinoma and 66/120 (55%) of invasive ductal carcinoma (Table 1, p < 0.05, χ2-test).

**Table 1:** The expression of HERC4 in normal breast tissues and in a series of breast tumors*

	**HERC4 protein**		
	**All cases**	**Negative expresion(%)**	**Positive expresion(%)**
Normal breast	16	15(93.7)	1(6.3)
Fibroadenoma	13	10(76.9)	3(23.1)
Intraductal carcinoma	15	9(60)	6(40)
Invasive ductal carcinoma	120	54(45)	66(55)

*Values are n (%). A significant increasing frequency of positive expression of HERC4 was detected in fibroadenoma, intraductal carcinoma, and invasive ductal carcinoma compared to adjacent normal breast tissue (P = 0.001, χ2-test).

### Association of HERC4 protein expression with clinicopathological parameters

The rates of positive expression of HERC4 with respect to usual clinicopathological parameters were shown in Table 2. The results demonstrated HERC4 was expressed increasingly in patients with advanced clinical stage (p < 0.001) and positive lymph node metastasis (p < 0.001). Moreover, HERC4 expression was positively correlate with pT status and histological grade (p < 0.001). There was no significant difference between HERC4 expression and other features, such as age (≤50 years vs >50 years) or ER, PR, Her2, p53, Ki67 status (P > 0.05).

**Table 2:** Association of HERC4 expression with patient’s clinicopathological features in 120 invasive ductal carcinoma

**Variables**	**HERC4 staining**			
	**Negative(%)**	**Positive(%)**	**Total**	**P value**^**b**^
Age(years)				
≤50^a^	25(45.5)	30(54.5)	55	0.927
>50	29(44.6)	36(55.4)	65	
pT status				
T1	15(88.2)	2(11.8)	17	0.000
T2	36(42.9)	48(57.1)	84	
T3	3(18.8)	16(81.2)	19	
Lymph node metastasis				
Negative	32(80)	8(20)	40	0.000
Positive	22(27.5)	58(72.5)	80	
Histology grade				
I	41(95.3)	2(4.7)	43	0.000
II	13(24.5)	40(75.5)	53	
III	0(0)	24(100)	24	
Stage				
I-II	43(76.8)	13(23.2)	56	0.000
III-IV	11(17.2)	53(82.8)	64	
ER status				
Negative	25(43.1)	33(56.9)	58	0.686
Positive	29(46.8)	33(53.2)	62	
PR status				
Negative	31(50)	31(50)	62	0.255
Positive	23(39.7)	35(60.3)	58	
HER2 status				
Negative	15(42.9)	20(57.1)	35	0.762
Positive	39(45.9)	46(54.1)	85	
P53 status				
Negative	23(41.8)	32(58.2)	55	0.519
Positive	31(47.7)	34(52.3)	65	
Ki67 status				
Negative	15(38.5)	24(61.5)	39	0.318
Positive	39(48.1)	42(51.9)	81	

^a^ Mean age.

^b^ P value are from χ2-test.

## Discussion

To date, etiology of breast cancer are unclear. Although several key genes are identified to be mutated or deregulated in breast cancer such as HER2, P53, cyclin E, and BRCA1/2 which are involved in tumorigenesis, progression and metastasis [22,23], the novel molecular markers are urgently needed for identifying tumor spread and aiding risk assessment. Last several years, ubiquitin-proteasome pathway has been seen as a key process in tumorigenesis [15]. HERC4 is recently identified as a member of HERC family and possesses ubiquitin ligase activity. Murine HERC4 specifically degrades spermatogenesis-related proteins by ubiquitin-proteasome pathway. Disfunction of murine HERC4 would prevent spermatozoon from maturation and influence male fertility [21]. To the best of our knowledge, the relationship between human HERC4 expression and tumorgenesis has not been reported so far. Here we study whether HERC4 expression exsit between breast cancer and adjacent normal breast tissues and its clinicopathological significance in patients with invasive ductal carcinoma.

Breast cancer cell lines are preclinical models that represent different breast tumor subtypes to some extent and that studies performed with these cell lines can be transitted to primary breast tumors. Therefore, we detected the HERC4 expression in breast cell lines by western blot analysis. The result showed that HERC4 was up-regulated in nearly all breast cancer cell lines compared to non-tumorigenic breast epithelial cell line MCF-10A, suggesting higher expression of HERC4 exists in tumor cells of clinical breast cancer samples. To validate our inference, we checked HERC4 expression in 32 paired breast cancer (13 intraductal carcinoma and 19 histologically confirmed invasive ductal carcinoma) and adjacent normal breast tissue samples by quantitative real-time PCR and western blot analysis. The results were in line with expectations that expression of HERC4 is significantly higher in the breast cancer cells than in the adjacent normal breast cells whether on mRNA level or on protein level (P < 0.05).

Furthermore, to assess clinicopathological significance of HERC4, we performed immunohistochemical analysis on a tissue microarray containing 13 benign fibroadenoma, 15 intraductal carcinoma, 120 histologically confirmed invasive ductal carcinoma and 16 adjacent normal breast tissue samples. We introduced a scoring system method based on percentage of positive staining tumor cells to assess HERC4 immunoreactivity. In order to avoid arbitrary cutpoints for IHC evaluation of HERC4, ROC analysis was applied to select optimal cut-off point for HERC4 positivity. Various ROC curves were plotted according to clinicopathological parameters, including pT stage, pN stage, clinical stage, histological grade and ER, PR, Her2, p53, Ki67 status. Finally, the cut-off score was selected to be above 60% for HERC4 positive expression. On the basis of cut-off point, positive expression is in 1/16 (6.3%) of normal breast tissue, in 3/13 (23.1%) of fibroadenoma, in 6/15 (40%) of intraductal carcinoma and 66/120 (55%) of invasive ductal carcinoma (p < 0.05). Positive expression of HERC4 was positively correlated with invasive ductal carcinoma pT status, pN status, clinical stage and histological grade (p < 0.05). These results suggest upregulation of HERC4 in breast cancer promote invasion and/or metastasis of cancer cells.

Taken together, our result indicated that HERC4 could be used as a significant diagnostic marker for invasive ductal carcinoma. As for the mechanisms of HERC4 potential regulation of breast cancer cells invasion and migration need further investigation.

## Conclusions

HERC4 is up-regulated in breast cancer cell lines and breast tumor tissues. Positive expression of HERC4 is positively correlated with pT status, pN status, clinical stage and histological grade of patients with invasive ductal carcinoma of breast (p < 0.05), which suggest that HERC4 may be a potential oncogene of breast cancer and a significant diagnostic marker for invasive ductal carcinoma.

## Methods

### Cell lines and cultures

Human non-tumorigenic breast epithelial cell line MCF-10A, human breast cancer cell lines MCF-7, MDA-MB-231, T-47D, SK-BR-3 and BT-474 were obtained from laboratory preservation. MCF-10A was grown in Dulbecco’s modified Eagle’s medium/F12 medium (15 mM hepes buffer, Hyclone, U.S.A.) containing 5% (vol/vol) donor equine serum, 10 μg/ml insulin, 20 ng/ml epidermal growth factor, 500 ng/ml hydrocortisone, 100 ng/ml cholera toxin, 100 units/mL penicillin and 100 μg/mL streptomycin (Hyclone, U.S.A.). Five human breast cancer cell lines were cultured in Dulbecco’s modified Eagle’s medium (DMEM High Glucose, Hyclone, U.S.A.) supplemented with 10% (vol/vol) fetal bovine serum (Gibco, U.S.A.), 100 units/mL penicillin and 100 μg/mL streptomycin (Hyclone, U.S.A.). All cell lines were cultured at 37°C in a 5% CO_2_-humidified atmosphere.

### Patients and tissue samples

In this study, a total of 148 paraffin-embedded tissue samples from patients with breast tumors who received no prior treatments were enrolled for immunohistochemistry. Moreover, another 32 fresh paired breast cancer (13 intraductal carcinoma and 19 histologically confirmed invasive ductal carcinoma) and adjacent normal breast tissue samples were collected for quantitative real-time PCR and western blot analysis. All the samples were collected from Nanfang Hospital and Zhujiang Hospital of Southern Medical University between 2005 and 2012. The paraffin-embedded tumor cases included 13 benign fibroadenoma, 15 intraductal carcinoma and 120 histologically confirmed invasive ductal carcinoma. In addition, 16 adjacent normal tissue samples were added as control group. Clinicopathologic information of patients included age at diagnosis, pT status, lymph node metastasis, clinical stage, histology grade and IHC results of Her-2, ER, PR, p53, and Ki67. Ages of the 120 patients with invasive ductal carcinoma ranged from 26 to 71 years (mean, 50 years) and detailed data were shown in Table 2. The histologic grade was assessed according to Bloom–Richardson classification. This study was performed under a protocol approved by the Ethic Committee of the Nanfang Hospital with written informed consent of all patients for research.

### RNA isolation, reverse transcription and real-time PCR

Quantitative real-time PCR was adopted to relatively quantify and assess the mRNA expression of HERC4 in clinical samples. Total RNAs were isolated from fresh breast tissues using RNAiso Plus (Takara, China) and dissolved in nuclease free water. The RNA concentration and purity were measured by Biophotometer plus (Eppendorf, Germany). Besides, RNA samples were analysed for following procedures only when OD A260/A280 ratio was between 1.8 and 2.0. First-strand cDNAs were synthesized from qualified mRNAs by using PrimeScript® RT reagent Kit (Takara, China) according to product manual. Quantitative real-time PCR were performed by using the SYBR® Premix Ex TaqTM II Kit (Takara, China) in an ABI 7500 real-time PCR amplifier (Applied Biosystems, U.S.A.). The primer sequences were listed in Table 3.The PCR conditions were as follows: 95°C for 30 s, 40 cycles of amplification at 95°C for 5 s and 60°C for 34 s, followed by additional dissociation stage for testing reaction specificity. Each PCR reaction was repeated 3 times for stable results. Expression of HERC4 was normalized with respect to GADPH and fold changes were analyzed using the Comparative Ct (ΔΔct) method.

**Table 3:** Primers used in real-time PCR

**Primer ID**	**Primer sequences(5′-3′)**
HERC4-Forward	TGATAGATGGGGGATTGTCG
HERC4-Reverse	ACCCAGTGATTGGTGCTCAT
GAPDH-Forward	CTGGGCTACACTGAGCACC
GAPDH-Reverse	AAGTGGTCGTTGAGGGCAATG

GAPDH: Glyceraldehyde-3-phosphate dehydrogenase, as internal control.

### Western blot

Total proteins were extracted from cell lines and breast tissue samples using Radio-Immunoprecipitation Assay (RIPA) buffer containing 1 mM PMSF (Beyotime, China). Then protein concentration was determined by BCA Protein Assay Kit (Beyotime, China) and adjusted to a final concentration of 5 μg/ μL using the RIPA buffer. The protein samples were mixed with the loading buffer (Beyotime, China) in a volume ratio of 4 to 1 and were boiled for 5 min for denaturalization. Finally, 10 μL mixture of each sample was loaded for 10% SDS-PAGE gel electrophoresis and then transferred on a polyvinylidene fluoride (PVDF) membrane by a Semi-Dry transfer machine (Trans-blot SD, BioRad, USA). The membrane was blocked by 5% nonfat milk dissolved in Tris-Buffered Saline and Tween 20(1 X TBST) solution at room temperature for 2 h. Afterwards the membrane was immediately separately incubated with HERC4 primary antibody (Rabbit polyclonal, 1:1000, PL laboratories, U.S.A.) and β-actin primary antibody (Mouse monoclonal, 1:2000, NeoBioscience, China) at 4°C overnight. The membrane was washed by 1X TBST for 3 times and then incubated with corresponding HRP conjugated-secondary antibodies (Goat anti-Rabbit IgG, 1:5000, EarthOx, U.S.A.; Goat anti-Mouse IgG, 1:5000, NeoBioscience, China) at room temperature for 1 h, followed by 1 X TBST washing 3 times. Eventually, immunoreactive bands were visualized by using the BeyoECL Plus Kit (Beyotime, China) and figures were scanned by the Image Station 4000R PRO instrument (CareStream Health, U.S.A.). Protein bands were quantified as optical density value using Gel-Pro analyzer (Media Cybernetics, U.S.A.) and β-actin was set as internal control.

### Tissue microarrays (TMA) construction and immunohistochemistry

Tissue microarray was constructed according to standard method. Briefly, duplicate 0.6 mm diameter cylinders were punched from representative areas of tumor or normal tissue blocks (donor tissue block). Next, cylinders were re-embeded into a new paraffin block (recipient paraffin block) at predefined positions using a tissue-arraying instrument (Minicore, Mitogen, U.K.). Finally, 5-μm sections were cut from TMA block for immuohistochemistry analysis.

Immunohistochemical staining was carried out according to standard procedures. Formalin-fixed and paraffin-embedded tissue array was immersed in xylene for deparaffinization and in graded ethanol for rehydration. Before antigen retrieval treated with sodium citrate buffer (pH 6.0) at 100°C for 20 min, activities of endogenous hydrogen peroxidase were inhibited by 0.3% H_2_O_2_. Subsequently, tissue array was incubated with 5% normal goat serum for blocking, followed by incubation with HERC4 primary antibody (Rabbit polyclonal, 1:200, PL laboratories, U.S.A.) at 4°C overnight. After 3 times of washing by 1X PBS, tissue array was incubated at room temperature for 30 min with polymer peroxidase-labeled secondary antibody (Zhongshan biotech, China). Finally, counterstaining was performed by hematoxylin. Signal was visualized by using DAB Horseradish Peroxidase Color Development Kit (Beyotime, China). Phosphate-buffered saline replaced anti-HERC4 antibody as a negative control.

### IHC evaluation

Immunohistochemical expression of HERC4 was scored by three authors independently using a semi-quantitative scoring method. It was based on the evluation of percentage of positive tumor cells over the total tumor cells. The scores were expressed as 5% increments (0, 5%, 10%…100%). When difference appeared among the three authors, results were re-estimated until a consensus was reached.

### Selection of cut-off score

Receiver operating characteristic (ROC) curve analysis was introduced to reach an optimal cutoff score for tumor with increased HERC4 expression using 0,1-criterion. For ROC analysis, the clinicopathological parameters were dichotomized as follows: pT stage (T1-T2, T3-T4), pN stage (N0, N1), clinical stage (I-II, III-IV), histological grade(G1-G2, G3) and ER, PR, Her2, p53, Ki67 status (negative, positive). ROC curves were generated by plotting the paired sensitivity and specificity of different HERC4 scores. The score closest to the point [0.0, 1.0] on the curve (i.e. the point with both maximum sensitivity and specificity) was treated as the cut-off score. Thus, tumors were defined “negative” when HERC4 score below the threshold. On the contrary, when HERC4 score was above the threshold, tumors was defined “positive”.

### Statistical analysis

All statistical analyses was performed with SPSS 13.0 software (SPSS, Chicago, IL, USA). Measurement data were showed as the mean ± SD. T test were used to assess expression differences within groups. Receiver operating characteristic (ROC) was applied to reach optimal cut-off score for HERC4 positive. The association between HERC4 expression and the clinicopathological parameters of the breast cancer patients was estimated with Chi-square test. Differences were considered significant when the P-value was <0.05 (two-tailed test).

## Competing interests

The authors declare that they have no competing interests.

## Authors’ contributions

HZ, RS carried out the molecular, immunological experiments and drafted the manuscript. WLM, JYZ participated in the design of the study and coordination. RS, WM, WLZ performed the statistical analysis. All authors read and approved the final manuscript.
